# Outcomes After Laparoscopic Radical Prostatectomy Versus da Vinci Robot-Assisted Laparoscopic Radical Prostatectomy

**DOI:** 10.7759/cureus.105060

**Published:** 2026-03-11

**Authors:** Carlos Tejeda-Andrade, Said Castro-Zazueta, Mario Eduardo Galland-Novelo, Francisco Gomez-Regalado, Alejandro Figueroa-Garcia, Leonardo Israel Ruiz-Guerrero, María Luisa Vazquez-Villegas

**Affiliations:** 1 Urology, Hospital Angeles del Carmen, Guadalajara, MEX; 2 Urology, Hospital General Dr. Agustín O'Horán, Mérida, MEX; 3 Epidemiology, Unidad Medica Familiar 4, Instituto Mexicano del Seguro Social, Guadalajara, MEX

**Keywords:** laparoscopic radical prostatectomy, laparoscopic technique, prostate cancer, radical prostatectomy, robot-assisted radical prostatectomy, robotic surgical procedures

## Abstract

Purpose

The study aimed to compare perioperative outcomes, early biochemical results, and recovery of urinary continence following laparoscopic radical prostatectomy (LRP) with da Vinci robot-assisted LRP (RALRP) in a group of men with favorable low- and intermediate-risk localized prostate cancer at a single hospital center.

Materials and methods

In this retrospective longitudinal study, we analyzed the clinical data of 48 patients with prostate cancer. Among them, 34 intervened with LRP and 14 with RALRP using the da Vinci system at Angeles del Carmen Hospital in Guadalajara, Mexico, from March 2023 to July 2025. The variables included were patient demographics and clinical and surgical data. Statistical analyses comprised both descriptive and parametric tests.

Results

Median age was 66 years. RALRP demonstrated longer operative time (198 min vs. 129 min, p≤0.001) and more blood loss (100-500 mL, 88% vs. 64% in RALRP, p=0.02). Continence recovery at 3 months was 70% after RALRP, compared with 65% after LRP and >90% at 12 months in both groups. At six-month follow-up, 95% had a prostate-specific antigen (PSA) level <0.2 ng/mL; no patient required salvage therapy.

Conclusions

In our study, both surgical techniques yielded good oncological results, with patients in both groups showing PSA levels indicating cure. However, continued follow-up of the population and an increased sample size are necessary.

## Introduction

Radical prostatectomy (RP) is a cornerstone treatment for localized prostate cancer. Minimally invasive approaches are widely used, yet evidence remains mixed regarding the superiority of laparoscopic RP (LRP) or robot-assisted LRP (RALRP). Some studies report perioperative advantages of robotic surgery, while oncologic benefits remain similar across techniques [1]. 

Prostate cancer is one of the most frequently diagnosed cancers in men. The global incidence rate of prostate cancer is estimated at approximately 29.4 cases per 100,000 men per year, according to Globocan (2022) [1]. Management encompasses a range of treatment options, including active surveillance, radiation therapy, and surgical interventions. Among these, RP is a prominent choice for localized disease treatment that involves the complete removal of the prostate gland. Complications of this procedure include urinary incontinence at 12 months (5%-20%); persistent severe incontinence (2%-5%), the most frequent functional complication; erectile dysfunction (30%-70%); significant bleeding (1%-5%); need for transfusion (0.5%-4%); and vesicourethral anastomosis stenosis (1%-5%) [2].

The definition of a biochemical “cure” using a prostate-specific antigen (PSA) level <0.2 ng/mL is under ongoing evaluation, given new evidence questioning its prognostic significance [3]. According to Kim et al., a recent network meta-analysis encompassing over 80 studies found that RALRP was associated with a significantly lower rate of positive surgical margins than open RP (relative risk: 0.893 and 95% credible interval: 0.807-0.985), suggesting improved local oncologic control with the robotic approach [3]. In contrast, LRP did not demonstrate a statistically significant difference in margin status compared with open surgery. LRP and RALRP are minimally invasive surgical techniques for the treatment of localized prostate cancer. LRP uses small keyhole incisions and an endoscopic camera to guide surgical dissection, which minimizes tissue trauma compared with traditional open prostatectomy [3]. The minimally invasive approach, including both LRP and RALRP, has been associated with reduced intraoperative blood loss, shorter hospital stays, and faster postoperative recovery, with improved perioperative outcomes and accelerated functional recovery relative to open surgery. 

Recent advancements in surgical techniques, particularly LRP and RALRP, have demonstrated several advantages over traditional open surgery. For instance, a study by Kim et al. emphasizes that laparoscopic and robotic approaches result in reduced blood loss, shorter hospital stays, and faster recovery times without compromising oncological outcomes [3]. Furthermore, robotic-assisted surgery offers enhanced precision and improved visualization, providing surgeons with greater control and potentially reducing postoperative complications. According to Wang et al., RALRP is associated with fewer overall complications than the open technique, as well as less bleeding and a lower need for transfusion [4].

RALRP, an advancement of LRP, uses robotic systems to enhance three-dimensional visualization and surgical precision, facilitating delicate dissection and preservation of surrounding structures such as neurovascular bundles. This enhanced precision may contribute to improved postoperative functional outcomes, including greater preservation of erectile function compared with conventional approaches [5].

A technical assessment is relevant because it reflects real-world oncological outcomes in countries where laparoscopy is the predominant approach and robotic surgery is an initial implementation phase, providing documentation on feasibility, oncological safety, and technological transition, as well as useful information for other healthcare systems with similar characteristics.

Therefore, the aim of this study was to compare perioperative outcomes, early biochemical outcomes, and recovery of urinary continence between LRP and RALRP with the da Vinci system in men with favorable, low- and intermediate-risk localized prostate cancer. Additionally, we sought to evaluate whether the robotic approach provides any potential advantages over conventional laparoscopy in terms of surgical safety, early oncological control, and functional recovery in a real-world clinical setting. All procedures were performed at a single tertiary-care hospital, allowing for a consistent surgical environment and standardized perioperative management.

## Materials and methods

Design

This was a retrospective longitudinal study.

Setting

Patients with prostate cancer at low and favorable intermediate risk (of aggressive disease and/or metastasis or progression) were included. In this retrospective longitudinal study, we analyzed the clinical data of 48 patients with prostate cancer. Among them, 34 were intervened with LRP and 14 with RALRP using the da Vinci system at Angeles del Carmen Hospital in Guadalajara, Mexico, from March 2023 to July 2025. The variables included were patient demographics and clinical and surgical data. Statistical analyses comprised both descriptive and parametric tests.

Selection criteria

The inclusion criteria were patients with: a) low surgical risk (cT1-cT2a), grade group one, and PSA <10 ng/dL, and b) favorable intermediate risk (cT2b-cT2c), grade group one or two, <50% biopsy cores positive, and PSA 10-20 ng/dL (National Comprehensive Cancer Network. (2025). Prostate Cancer (version 4.2026) [6]. No exclusion criteria were applied.

Clinical evaluation

One trained researcher used a structured clinical chart containing data on epidemiological and clinical variables and surgical intervention.

Surgical procedure

The surgical technique was performed by the same surgeon with more than 10 years of experience in LRP and more than 5 years in RALRP. All surgical procedures were performed using the same standard intraperitoneal technique for RP, starting from the bladder neck and proceeding to the prostatic apex. A nerve-sparing procedure (intrafascial, unilateral, or bilateral) was performed after consultation with the patient, based on multiparametric magnetic resonance image results, risk classes, risk of extracapsular disease, and the likelihood of maintaining potency. Pelvic lymphadenectomy was performed in patients with a >5% risk according to predictive nomograms.

Statistical analysis

We expressed quantitative variables as means and standard deviations, while qualitative data were presented as frequencies (%). Comparisons of means used the Student t-test, and the Pearson chi-square test analysis was used for comparisons of frequencies. The cut-off for statistical significance was p≤0.05. Statistical analysis was performed using the Statistical Package for Social Sciences (SPSS) version 20 (IBM Corp., Armonk, NY).

## Results

Table 1 shows the general characteristics of the study patients. The average age was 66 years (range: 50-82 years), the average PSA was 7.3 ng/mL, 71% had T1c tumors, and the median number of positive samples was five. After the surgical intervention, PSA decreased to a mean of 0.02 ng/mL, and incontinence occurred only in two patients who had it before the surgery.

**Table 1:** Description of clinical features and surgical characteristics of the patients Qualitative data are presented as frequencies; quantitative variables are expressed as means ± standard deviations (SDs).

Variable	n=48
Age (years), mean ± SD	66 ± 8
Prostatic antigen (ng/mL), mean ± SD	7.3 ± 2.3
Gleason score, mean ± SD	7 ± 1
Lateral, n (%)	
Right	20 (40)
Left	14 (30)
Both	14 (30)
Suspicious rectal examination	16 (33)
Tumor-nodes-metastasis (TMN), n (%)	
T1a	2 (4)
T1b	5 (10)
T1c	34 (71)
T2a	4 (8)
T2b	1 (2)
T2c	2 (4)
Pelvic lymphadenectomy, n (%)	25 (52)
Number of samples, mean ± SD	12 ± 1
Number of positive samples, mean ± SD	5 ± 2
Incontinence, mean ± SD	2 ± 1
Prostatic antigen follow-up, mean ± SD	0.04 ± 0.05
Bleeding (mL), n (%)	
<100	2 (4)
100-500	39 (81)
501-1000	7 (15)
Surgery time (min), mean ± SD	149 ± 55

Table 2 compares the two groups that received RP. A total of 34 patients underwent LRP, and 14 underwent RALRP. The RALRP group had a younger population than the LRP group (68 vs. 61 years, p=0.002).

**Table 2:** Comparison of clinical features and surgical characteristics between laparoscopic radical prostatectomy and robot-assisted laparoscopic prostatectomy Qualitative data are presented as frequencies (%); quantitative variables are expressed as means ± standard deviations (SDs). Proportions were compared with the chi-square test, and means were compared with the Student t-test.

Variable		Laparoscopic radical (n=34)	Robot-assisted (n=14)	Chi-square* or t-value**	p-value
						Age (years), mean ± SD		68 ± 7	61 ± 6	3.22**	0.002
						Prostatic antigen, mean ± SD		7.2 ± 1.8	7.6 ± 3.3	-0.57**	0.56
Gleason score, mean ± SD		6.5 ± 1	6.6 ± 1	-0.70**	0.48
Laterality, n (%)					
Right		13 (38)	7 (50)		
Left		10 (30)	4 (29)	0.73*	0.69
Both		11 (32)	3 (21)		
Suspicious rectal examination, n (%)		13 (38)	3 (21)	1.26*	0.26
Tumor-nodes-metastasis (TMN), n (%)					
T1a		2 (6)	-		
T1b		5 (15)	-		
T1c		20 (58)	14 (100)	8.13*	0.14
T2a		4 (12)	-		
T2b		1 (3)	-		
T2c		2 (6)	-		
Pelvic lymphadenectomy, n (%)		16 (47)	9 (64)	1.17*	0.27
Number of samples, mean ± SD		12 ± 2	11 ± 1	0.53**	0.59
Number of positive samples, mean ± SD		5 ± 2	5 ± 1	-0.35**	0.72
Incontinence, mean ± SD		2 ± 1	2 ± 1	-0.44**	0.66
Prostatic antigen follow-up, mean ± SD		0.04 ± 0.04	0.05 ± 0.05	0.03**	0.97
Bleeding (mL), n (%)					
<100		2(6)	-		
100-500		30 (88)	9 (64)	7.57*	0.02
501-1000		2 (6)	5 (36)		
Surgery time (min), mean ± SD		129 ± 37	198 ± 62	-4.84**	≤0 .001

The following variables did not show statistically significant differences: initial PSA, Gleason score, lateral (right 20/40%, left 14/30%, and both 14/30%), suspicious rectal examination, TNM classification, pelvic lymphadenectomy, positive samples, and complications after surgery. All patients presented PSA levels indicating healing (<0.02 mg/dL) at follow-up four to six weeks after the surgery.

Bleeding tended to be lower in LRP (88% (100-500 mL) vs. 64% in RALRP, p=0.02), while operative time was longer in RALRP (198 minutes vs. 129 minutes in LRP, p≤0.001).

## Discussion

In this study, we observed that RALRP required a longer operative time and resulted in more bleeding than LRP. In a cohort study, Salciccia et al. reported that the mean operating time was significantly shorter with RALRP than with laparoscopic prostatectomy, whereas our work showed that the operating time was significantly longer for robotic prostatectomy [7]. No differences were observed in the majority of variables; both procedures showed favorably reduced PSA levels after surgery.

According to Kim et al., there is no significant difference in estimated blood loss between RARP and LRP, as reported in our results.

Following RP, PSA levels are expected to decrease to undetectable levels, reflecting the complete removal of prostate tissue. Biochemical cure is conventionally defined as a postoperative PSA level below 0.2 ng/mL, a widely accepted threshold for distinguishing between successful oncological control and biochemical recurrence. Recent evidence suggests that the use of ultrasensitive PSA (uPSA) assays provides additional prognostic value, particularly for long-term outcomes. Kawamura et al. demonstrated that persistently undetectable or very low uPSA levels after surgery were strongly associated with a significantly lower risk of late biochemical recurrence, supporting the concept that PSA levels well below 0.2 ng/mL represent durable biochemical remission and can be considered indicative of curative treatment in appropriately selected patients, as demonstrated in the postoperative outcome of the entire population in our study [8]. This reinforces the clinical relevance of a PSA <0.2 ng/mL not only as a definition of non-recurrence but also as an indirect marker of oncological cure after RP.

In Latin America, the implementation of RALRP remains limited by cost, infrastructure, and availability, restricting its use to select centers with a high volume of patients. Consequently, LRP continues to be the most viable minimally invasive approach in the region. As reported by Rosino et al., oncological outcomes between laparoscopic and robot-assisted techniques are comparable, supporting the continued role of laparoscopy in resource-limited settings [9]. Consistent with this regional reality, our institutional experience reflects a predominant use of LRP, with 34 patients treated laparoscopically compared with 14 undergoing robotic surgery. These data underscore the practicality and sustainability of laparoscopy as the primary minimally invasive technique, while robotic surgery continues to be applied selectively where resources allow.

According to Wang et al., several systematic reviews and large observational studies have reported that minimally invasive RP (robot-assisted or laparoscopic) is associated with substantially reduced estimated blood loss and transfusion requirements, as well as lower overall complication rates compared with open RP [4]. For example, robot-assisted approaches have demonstrated a 77% lower probability of requiring perioperative blood transfusion, significantly less blood loss (~516 mL reduction), and reduced overall complications (OR: ~0.61) compared to open surgery. According to Pessoa et al., from a functional perspective, robotic surgery is associated with better rates of urinary continence at 12 months [10]. Several comparative analyses have reported better urinary continence results after minimally invasive RP, similar to the results of our study, with the difference that, in our population, only two patients who already had incontinence before surgery presented with it. For example, pooled data indicate that patients undergoing RALRP have lower rates of postoperative urinary incontinence at 12 months compared with open RP (7.5% vs. 11.3%, respectively), which suggests a functional advantage of the minimally invasive approach. Oncologically, a recent network meta-analysis comparing robotic, laparoscopic, and open RP reported that RALRP had a significantly lower rate of positive surgical margins compared with open RP (relative risk: 0.893, 95% credible interval: 0.807-0.985) [3]. This finding indicates an oncologic advantage in local tumor control for the robotic approach, which is associated with a modest but significant decrease in early biochemical recurrence [3]. In contrast, all patients included in our sample presented PSA levels indicating healing. However, these differences are not reflected in significant improvements in disease-free, cancer-specific, or overall survival, underscoring that surgeon experience and institutional volume remain the determining factors for long-term oncological outcomes. PSA thresholds for defining cure should be interpreted with updated evidence.

The limitations of this study are inherent to the proposed epidemiological design. One limitation of this retrospective study is that it may have some information bias; however, this was considered during patient selection. Although the sample size is small compared with other studies, we believe that in our area, few patients have access to robotic procedures, primarily due to limited availability. Furthermore, prospective long-term follow-up will be necessary.

## Conclusions

In this retrospective comparative study, both techniques achieved similar oncologic and functional outcomes in patients with low and favorable intermediate risk prostate cancer. While the robotic approach may offer certain technical advantages, LRP remains a safe, effective, and oncologically sound alternative, particularly in settings where access to robotic platforms is limited. These findings support the continued role of laparoscopy in resource-constrained environments and highlight the importance of surgical expertise over technological modality. However, according to the available evidence, RALRP has demonstrated statistically significant superiority over LRP, primarily in perioperative and functional outcomes, as well as modest but consistent early oncological advantages. Further prospective and long-term studies are warranted to confirm these results.

## References

[1] Sung H, Ferlay J, Siegel RL, Laversanne M, Soerjomataram I, Jemal A, Bray F (2021). Global cancer statistics 2020: GLOBOCAN estimates of incidence and mortality worldwide for 36 cancers in 185 countries. CA Cancer J Clin.

[2] Kesch C, Heidegger I, Kasivisvanathan V (2021). Radical prostatectomy: sequelae in the course of time. Front Surg.

[3] Kim DK, Moon YJ, Chung DY (2025). Comparison of robot-assisted, laparoscopic, and open radical prostatectomy outcomes: a systematic review and network meta-analysis from KSER update series. Medicina (Kaunas).

[4] Wang J, Hu K, Wang Y (2023). Robot-assisted versus open radical prostatectomy: a systematic review and meta-analysis of prospective studies. J Robot Surg.

[5] Pina AJ, Melo VC, Carlos VW (2025). Erectile function after laparoscopic versus robotic-assisted radical prostatectomy: a systematic review and meta-analysis. Asian J Urol.

[6] (2026). National Comprehensive Cancer Network: NCCN guidelines. https://www.nccn.org/guidelines/guidelines-detail.

[7] Salciccia S, Santarelli V, Di Pierro GB (2024). Real-life comparative analysis of robotic-assisted versus laparoscopic radical prostatectomy in a single centre experience. Cancers.

[8] Kawamura N, Nakayama M, Hayashi T, Nagahara A, Nakai Y, Nishimura K (2025). Risk assessment of late biochemical recurrence after radical prostatectomy: usefulness of ultra-sensitive prostate-specific antigen measurement. Int J Urol.

[9] Rosino A, Pietricica B, Cívico-Sánchez C (2025). Comparative analysis of initial series: robot-assisted vs laparoscopic radical prostatectomy. Urología Colombiana.

[10] Pessoa RR, Maroni P, Kukreja J, Kim SP (2021). Comparative effectiveness of robotic and open radical prostatectomy. Transl Androl Urol.

